# YOLO-RMF: A Real-Time Surface Defect Detection Model for Hot-Rolled Strip Steel with Reparameterized Convolution and Axial Attention

**DOI:** 10.3390/s26206533

**Published:** 2026-10-15

**Authors:** Zhijun Rong, Aohan Deng, Yiheng Wang, Bingbing Dan, Liping Du, Chun Zhou

**Affiliations:** School of Mechanical Engineering, Wuhan University of Science and Technology, Wuhan 430081, China; rongzhijun@wust.edu.cn (Z.R.); yihengwang@lyricrobot.com (Y.W.); danbinbin@wust.edu.cn (B.D.); duliping@wust.edu.cn (L.D.); zhouchun@wust.edu.cn (C.Z.)

**Keywords:** hot-rolled strip steel, surface defect detection, reparameterized convolution, axial attention, edge computing

## Abstract

Hot-rolled strip steel surface defect detection is a vital industrial task that demands both high precision and real-time inference. Nevertheless, existing deep-learning-based detectors struggle to balance accuracy and speed, limiting their practical deployment on resource-constrained edge devices within harsh manufacturing scenarios. To address this gap, this paper develops YOLO-RMF, a real-time one-stage detector for hot-rolled strip steel inspection. Four core components are presented: a reparameterized 2 × 2 convolution (RPRC2) for enhanced feature extraction with lower inference computational cost than standard 3 × 3 convolutions, a two-dimensional axial attention (TDAA) module to capture long-range spatial dependencies, an adaptive-weighted multi-scale feature fusion pyramid network (MSF-FPN), and a hybrid adaptive pruning strategy combining BN scaling factors and feature-map rank analysis for model compression. Evaluated on NEU-DET and MVIT datasets, YOLO-RMF obtains 76.1% mAP50 and 40.6% mAP50-95 at 212 FPS (batch size 1, RTX 4090 GPU), achieving a favorable balance between detection sensitivity and precise localization for edge-computing-based industrial inspection. The pruning scheme reduces parameters by 36.3% and GFLOPs by 49.0% with minor accuracy loss. Deployed on Jetson AGX Orin, the pruned model reaches 169 FPS under FP16 and 233 FPS under INT8 quantization. Experimental outcomes show that YOLO-RMF achieves a favorable accuracy-speed trade-off and is suitable for practical industrial edge-based defect inspection scenarios.

## 1. Introduction

Hot-rolled strip steel represents one of the most fundamental materials in modern manufacturing, serving as a critical input for automotive, construction, shipbuilding and home appliance industries. The global production of hot-rolled strip steel exceeded 1.8 billion tons in 2025 [1], with surface quality directly affecting product value and downstream manufacturing processes. Surface defects such as scratches, roll marks, patches, and inclusions can reduce product grade by up to 30% [2], leading to significant economic losses for steel manufacturers. Traditional manual inspection methods suffer from low efficiency, high labor intensity, and inconsistent detection accuracy, with typical detection rates ranging from 60% to 75% even for experienced inspectors [3,4].

The development of computer vision technology has enabled automated surface inspection systems in steel production lines. Modern hot-rolling mills operate at speeds up to 25 m/s. Assuming that the camera’s field of view along the rolling direction is 125 mm, and that a spatial sampling interval of 0.1 mm without missed detections is to be achieved, the minimum detection frame rate required when the strip running speed is 25 m/s (25,000 mm/s) is as follows:

$$FPS_{\min}=\frac{v}{L_{FOV}}=\frac{25000}{125}=200FPS$$

This stringent real-time requirement, combined with the high variability of defect appearances, complex illumination conditions, and massive image data volumes, creates significant challenges for existing computer vision algorithms.

### 1.1. Problem Statement

**Figure 1:**
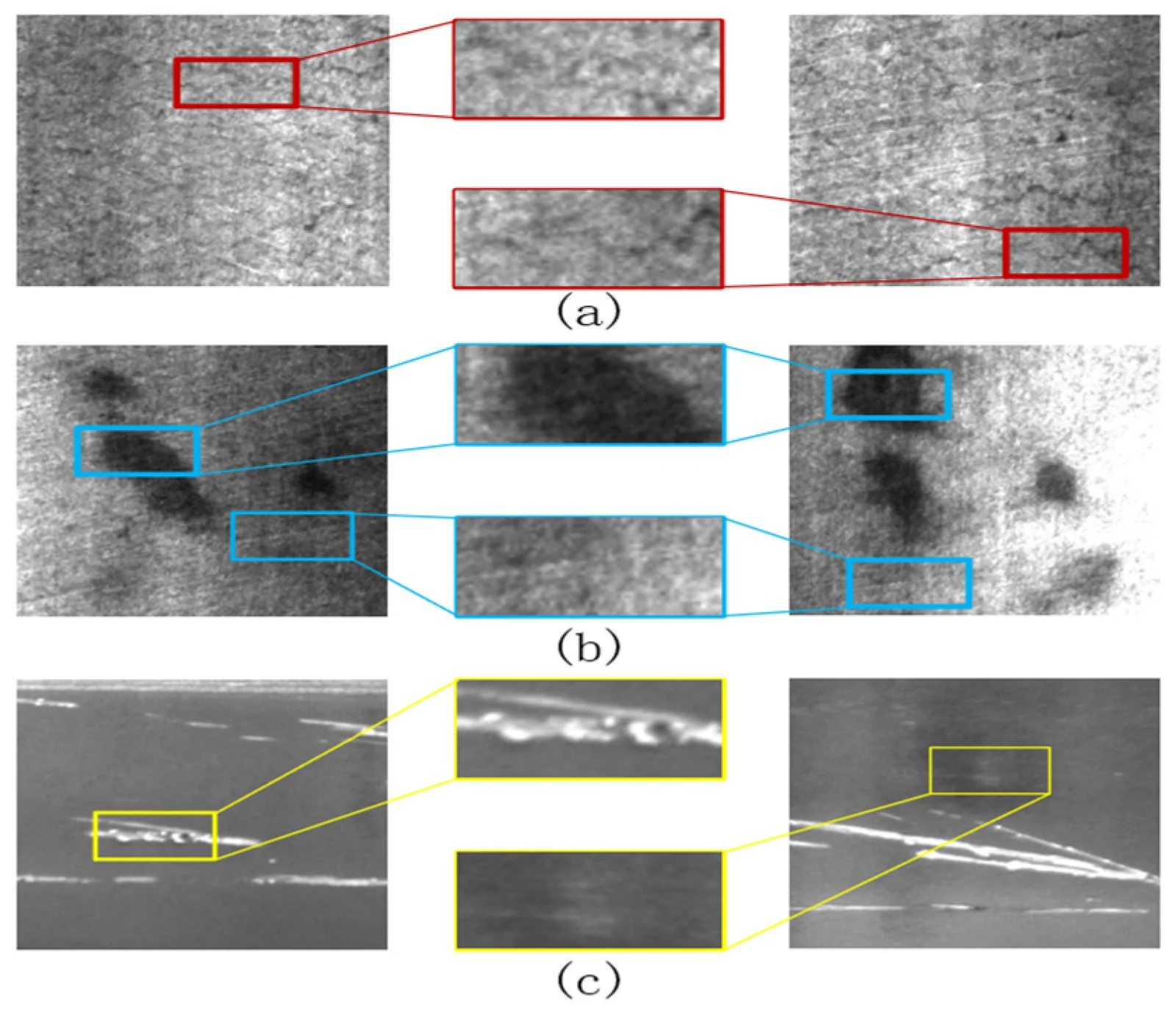
Challenges of defect inspection from industry. (**a**) Defects with low contrast and high noise. (**b**) Defects with extreme scale variation. (**c**) Defects with class imbalance.

Hot strip steel defect detection faces four unique industrial challenges distinct from general object detection:

1. Low-contrast and heavy noise: High-temperature steam, thermal radiation and oxide scales blur defect boundaries (Figure 1a).
2. Extreme scale variation: Defects range from 0.1 mm micro-scratches to meter-level large patches (Figure 1b).
3. Severe class imbalance: Frequent scratches vs. rare holes with an occurrence ratio below 0.1% (Figure 1c).
4. Edge deployment limits: Industrial embedded devices have tight memory, power and computing budgets.

Notably, the above descriptions reflect general characteristics of industrial hot-rolled strip production rather than the specific composition of the datasets used in this work. The NEU-DET dataset adopted in our experiments contains six representative defect categories (crazing, inclusion, patches, pitted surface, rolled-in scale, scratches), and the MVIT dataset covers multiple common defect patterns captured from real factory environments. We note that both benchmark datasets are approximately class-balanced: NEU-DET contains 300 images per class, and the test split preserves a balanced instance distribution across categories. Consequently, the class-imbalance challenge described above is not fully reflected in our experimental evaluation. The potential impact of this discrepancy on the interpretation of our results is discussed in Section 5.3.

### 1.2. Limitations of Existing Methods

Traditional computer vision methods based on handcrafted features (SIFT, HOG, LBP) [5] have been widely applied in industrial inspection but lack robustness to illumination variations and defect morphology changes. Convolutional neural network (CNN) based methods have demonstrated superior performance, with two-stage detectors such as Faster R-CNN achieving high accuracy but suffering from slow inference speeds unsuitable for real-time applications [6,7].

Single-stage detectors such as YOLO, SSD, and RetinaNet offer better speed-accuracy trade-offs but still face limitations in industrial deployment: The standard YOLOv8m variant requires over 50 GFLOPs for inference at 640 × 640 input resolution, exceeding the computational capacity of many edge devices [8] (in this work, we select the more lightweight YOLOv8n and YOLOv8s variants as baselines for fair comparison). Attention mechanisms such as Transformer architectures improve feature representation but introduce significant computational overhead, limiting their practical application in high-speed production environments.

Furthermore, existing defect detection models often require extensive computational resources during training and fail to provide systematic model compression strategies for edge deployment. The lack of end-to-end optimization from model design to deployment constitutes a significant barrier to practical industrial application [9,10,11].

### 1.3. Technical Challenges

This work addresses four core technical challenges in real-time surface defect detection:

1. **Speed-accuracy trade-off**: Extract low-contrast tiny defects without extra inference delay.
2. **Long-range dependency modeling:** Linear-complexity global spatial feature capture.
3. **Multi-scale fusion:** Jointly retain small-defect details and large-defect semantic features.
4. **Edge optimization**: Lightweight compression with negligible accuracy loss.

### 1.4. Contributions

This paper makes the following principal contributions:

1. RPRC2, a reparameterized 2 × 2 convolution block that strengthens low-contrast defect feature extraction. It fuses each convolutional layer with its subsequent batch normalization (BN) layer at inference to eliminate BN-specific runtime overhead, and achieves lower overall computational cost than a standard 3 × 3 convolution with equivalent receptive field.
2. TDAA axial attention decomposes 2D self-attention into axis-wise linear computation, reducing the quadratic complexity O(n^2^) (n = H × W) to O(HW(H + W)).
3. MSF-FPN adopts adaptive weighted bidirectional fusion to balance multi-scale feature information for tiny and large defects.
4. A hybrid adaptive pruning strategy combining BN scale factors and feature rank analysis compresses the model with minimal accuracy loss.
5. Comprehensive evaluations on NEU-DET and MVIT cover ablation, generalization, robustness, statistical significance and Jetson edge deployment verification.

## 2. Related Work

### 2.1. Surface Defect Detection Methods

Surface defect detection has evolved from traditional image processing techniques to modern deep learning approaches. Early work by Li [12] applied Gabor filters and support vector machines for texture defect classification, achieving acceptable accuracy for controlled laboratory conditions but failing in industrial environments. With the development of deep CNNs, transfer learning approaches gained popularity. Li et al. [13] adapted YOLOv5 architectures for steel surface defect classification, achieving 98.5% accuracy on six-class classification tasks. However, classification-only approaches cannot provide precise defect localization required for quality grading and root cause analysis. For object detection-based methods, Wang et al. [14] proposed an improved YOLOv8, achieving 82.3% mAP on NEU-DET but running at only 15 FPS, insufficient for real-time production.

Recent single-stage detector adaptations have shown promising results. Sun et al. [15] modified YOLOv5 with attention mechanisms for strip steel defect detection, achieving 85.5% mAP at 27.7 FPS. Du et al. [16] introduced Ghost convolution modules into AFF-Net for lightweight defect detection, reducing parameters by 40% but with 2.1% mAP degradation. While these approaches demonstrate progress, the fundamental speed-accuracy trade-off remains unresolved for high-speed hot-rolling applications.

### 2.2. Lightweight Object Detection Architectures

Lightweight network design has become a critical research direction for edge deployment. Li et al. [17] utilized depthwise separable convolution to reduce the computational cost by 8 to 9 times compared with standard convolution. Zhang et al. [18] introduced the lightweight convolutional layer GSConv, which reduces the number of network parameters and improves detection speed. However, direct application of these general-purpose lightweight architectures to defect detection often results in significant accuracy loss due to reduced feature extraction capability.

Structural reparameterization has emerged as a powerful technique for improving model performance without inference cost. Tang et al. [19] reparameterized the multi-branch structure into a single convolution, effectively reducing the time cost. You et al. [20] introduced structural reparameterization based on YOLOv5, merged the Neck and Head layers, and incorporated the RepVGG module to construct the Re-YOLOv5 model, significantly reducing inference time and computational overhead. These reparameterization techniques provide a theoretical foundation for the RPRC2 module proposed in this work.

Model pruning represents another essential approach for lightweight deployment. Ma et al. [21] combined pruning with knowledge distillation, enabling aggressive pruning with minimal accuracy loss. However, existing pruning methods often require extensive retraining and lack adaptability to specific defect detection characteristics.

### 2.3. Attention Mechanisms in Computer Vision

Attention mechanisms have revolutionized computer vision by enabling models to selectively focus on relevant information. Dosovitskiy et al. [22] demonstrated that capturing long-range dependencies significantly improves performance across vision tasks. Vision Transformers further established self-attention as a powerful alternative to convolution, achieving excellent results on ImageNet.

However, standard self-attention suffers from quadratic computational complexity with respect to input size, making it impractical for high-resolution industrial inspection images typically exceeding 2000 × 2000 pixels. Yu et al. [23] overcame this limitation by decomposing two-dimensional convolutions into axial one-dimensional operations, reducing computational complexity to a linear level while still capturing global dependencies. This approach has been successfully applied in medical image segmentation and image generation tasks.

In the defect detection domain, attention mechanisms have been applied primarily through squeeze-and-excitation networks and convolutional block attention modules [24,25]. While these channel and spatial attention modules improve feature discrimination, they operate within local receptive fields and cannot capture long-range spatial relationships essential for understanding defect context. The TDAA module proposed in this work extends axial attention specifically for defect detection, maintaining computational efficiency while providing global context modeling.

Although the foregoing studies have advanced surface defect detection from various perspectives, several research gaps still exist. First, existing reparameterization and attention techniques are generally designed for generic object detection and are not optimized for the low-contrast and high-noise properties of industrial defect images. Second, lightweight detectors frequently sacrifice feature extraction capacity to cut computational cost, which deteriorates performance on subtle defects. Third, few end-to-end optimization frameworks jointly handle architectural design, feature fusion and model compression for edge deployment in steel production. The proposed YOLO-RMF integrates customized reparameterized convolutions, axial attention, adaptive multi-scale fusion and hybrid pruning into a unified architecture to achieve real-time surface defect detection, and hopes to provide a feasible solution to fill the above research gaps.

## 3. Methodology

### 3.1. Overall Architecture of YOLO-RMF

**Figure 2:**
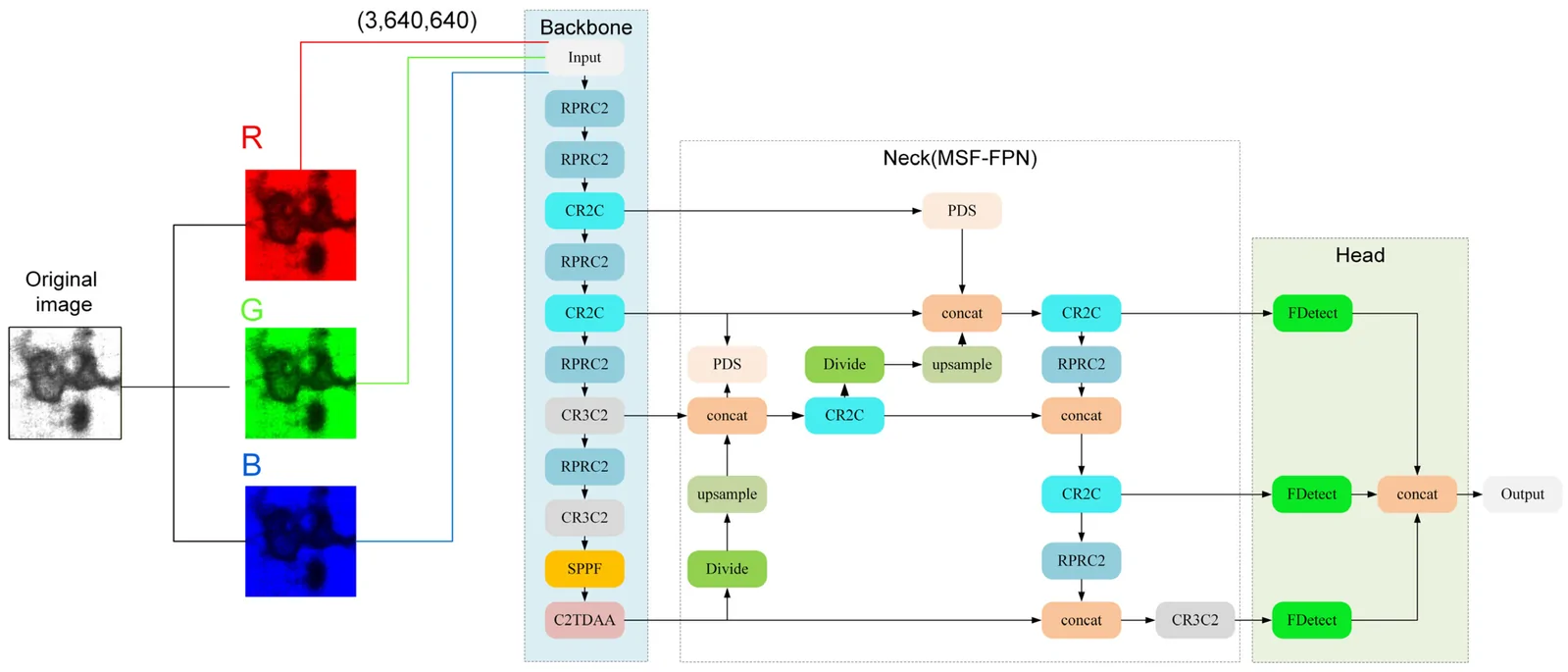
Architecture of the YOLO-RMF Model: The backbone network takes RGB three-channel defect images with a resolution of 640 × 640 pixels as input. After multi-stage feature extraction, it outputs four feature maps with shapes of (64, 160, 160), (128, 80, 80), (128, 40, 40), and (256, 20, 20). The downsampling ratios of the four feature maps are 4, 8, 16, and 32, respectively, which encode feature information corresponding to defects of various sizes on the input defect images. The Neck fuses the four feature maps of different sizes and passes the fused result to the Head. The Head computes the probabilities and locations of defects across all positions on the three feature maps based on the Neck’s output, then concatenates the detection results from these three feature maps to produce the final model output.

The YOLO-RMF architecture follows a single-stage detector design optimized for surface defect detection, consisting of four main components: backbone network, feature fusion neck, detection head, and optional pruning module. The overall architecture is illustrated in Figure 2.

The backbone network employs a Cross Stage Partial (CSP) Darknet-inspired design with RPRC2 modules replacing standard convolutions in early and middle stages. CR2C and CR3C2 modules provide cross-stage partial connections with enhanced gradient flow characteristics. TDAA modules are strategically inserted at the C3 and C4 feature levels to capture global spatial dependencies while maintaining computational efficiency.

The neck adopts the MSF-FPN design, integrating top-down and bottom-up feature fusion paths with adaptive feature weighting. Three detection levels (P3, P4, P5) process features at strides of 8, 16, and 32, corresponding to receptive fields optimized for small, medium, and large defects, respectively.

The FDetect detection head employs a cross-branch interactive design, sharing feature extraction parameters between classification and regression branches while maintaining separate prediction heads. This design improves feature utilization efficiency and reduces parameter redundancy.

After initial training, the adaptive pruning strategy analyzes BN scaling factors and feature map ranks to identify redundant channels, producing a compressed model suitable for edge deployment. The pruned model undergoes short-term fine-tuning to recover any accuracy loss resulting from structure removal.

### 3.2. RPRC2: Reparameterized 2 × 2 Convolution Module

**Figure 3:**
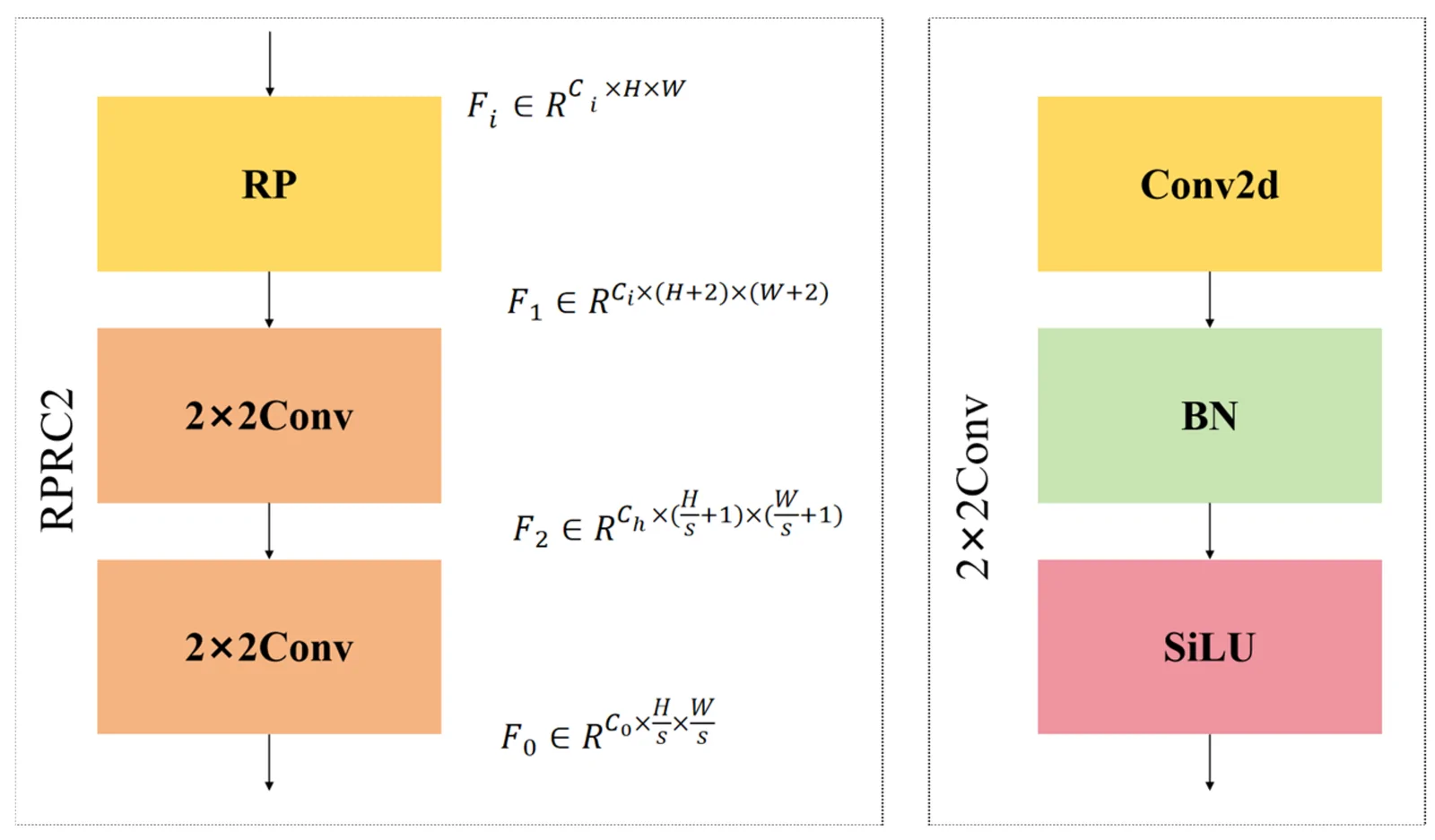
Architecture of the RPRC2 Block.

Structural reparameterization has been extensively investigated in prior literature, represented by RepVGG [20] and RepConv, where multi-branch training structures are fused into single-path inference architectures. Our RPRC2 module follows this principle and introduces three dedicated designs for low-contrast surface defect detection: (i) 2 × 2 kernel decomposition instead of the conventional 3 × 3 architecture, which reduces FLOPs by approximately 11% while maintaining an equivalent receptive field; (ii) replication padding rather than zero-padding to better preserve defect boundary information at feature-map edges and (iii) two-stage nonlinear activation with dual SiLU functions, strengthening the model’s capacity to learn complex feature mappings for subtle defect patterns. While the general reparameterization concept is adapted from prior work, the specific 2 × 2 design combined with replication padding is proposed for the first time in this paper and validated via ablation studies. Instead of conventional zero-padding, the module adopts Replication-Padding (RP), which effectively mitigates information attenuation of defect edge features during deep network propagation. Furthermore, a reparameterization mechanism is introduced to fuse the parameters of each convolutional layer (Conv2d) with its immediately following batch normalization (BN) layer at the inference stage. This fusion removes the separate forward computation of BN layers, simplifying the computation pipeline and reducing per-layer runtime overhead. Note that this reparameterization operates at the single-convolution level and does not merge the two cascaded convolutional stages, as the SiLU nonlinear activation between them prevents equivalent collapse into a single convolution. Even with two sequential convolutional layers, the total computational complexity of RPRC2 remains lower than that of a standard 3 × 3 convolution, as quantified in the following analysis. The detailed architecture of the RPRC2 model is illustrated in Figure 3.

Regarding the design of the RPRC2 module, two cascaded 2 × 2 convolutions reduce the feature map by two pixels along both the width and height dimensions. To preserve the original shape of feature maps, Replication-Padding is implemented prior to the convolution operation. After the replication padding step, two successive 2 × 2 convolutions are performed sequentially, whose calculation procedures are formulated in (1)–(4). Each 2 × 2 Conv block consists of a two-dimensional convolution (Conv2d) with kernel size *k* = 2, batch normalization (BN), and the SiLU activation function in sequence. Specifically, the stride s of the first 2 × 2 convolution is set to 2 to accommodate the requirement of multi-scale downsampling for feature maps of hot-rolled strip surface defects.

(1) 2×2Conv(X)=SiLU(BN(Conv2d(X/k=2)))

(2) $$C_{h}=\left\lfloor e\frac{max(C_{i},C_{o})}{2}\right\rfloor$$

(3) $$F_{o}=2\times 2Conv(2\times 2Conv(RP(F_{i}/p=1)s,C_{i},C_{h})/C_{h},C_{o},)$$

(4) $$F_{o}=RPRC2(F_{i}/C_{i},C_{o},s,e)$$

where *k* denotes the kernel size of the convolution; *C_i_*, *C_h_*, and *C_o_* denote the number of input channels, hidden channels, and output channels of the RPRC2 module, respectively; *e* denotes the channel scaling factor; *s* denotes the convolution stride; *F_i_* and *Fo* represent the input and output feature maps and *H* and *W* denote the height and width of the feature map.

With *s* = 1 and *e* = 1, the RPRC2 module has a computational complexity of O(8C^2^HW), approximately 11.1% lower than a standard 3 × 3 convolution (O(9C^2^HW)) while maintaining an equivalent receptive field. The two cascaded 2 × 2 convolutions embed two additional SiLU activations, strengthening the learning capacity for complex feature mappings of low-contrast defects. The channel scaling factor e further reduces computational cost when *e* < 1.

### 3.3. CR2C and CR3C2: CSP-Fused Feature Extraction Modules

**Figure 4:**
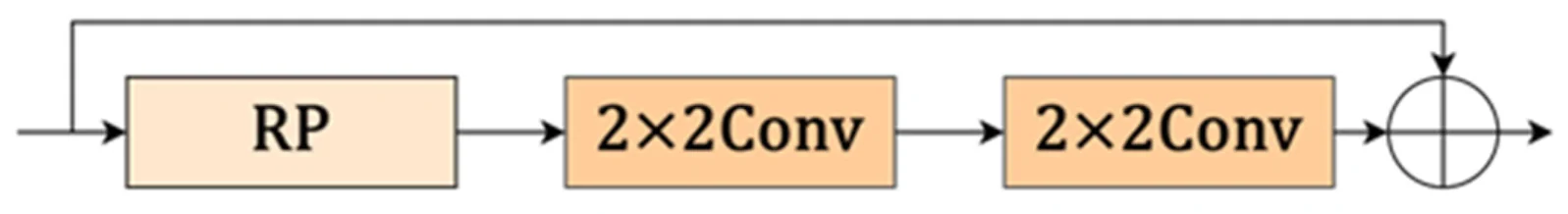
Architecture of the RPRC2-Res blocks.

The RPRC2 module efficiently realizes downsampling and channel dimension expansion of feature maps while preserving critical feature information, and it is mainly deployed in the downsampling layers of the backbone network. For non-downsampling layers, a variant of RPRC2 named RPRC2-Res is adopted. By integrating this variant with the Cross Stage Partial (CSP) architecture, two composite modules, namely CR2C (CSP-RPR with 2 convolutions) and CR3C2 (dual CR3C), are constructed as the core feature extraction units. Compared with the vanilla RPRC2 module, RPRC2-Res introduces a residual structure to optimize gradient propagation during training. Meanwhile, the stride of both cascaded 2 × 2 convolutions is set to 1 to prevent excessive spatial compression of feature maps. The architecture of RPRC2-Res is depicted in Figure 4.

For the surface defect detection task of hot-rolled strips, the discrimination between defects and backgrounds mainly relies on three types of features: color, texture, and spatial morphology characteristics. Nevertheless, the surface of hot-rolled strips generally presents a single color system such as silvery white or grayish black, resulting in low contrast between typical defects (e.g., cracks and scale indentations) and the steel background. This greatly raises the difficulty of extracting edge features of defects, and such a bottleneck severely restricts the improvement of detection accuracy for hot-rolled strip surface defects.

**Figure 5:**
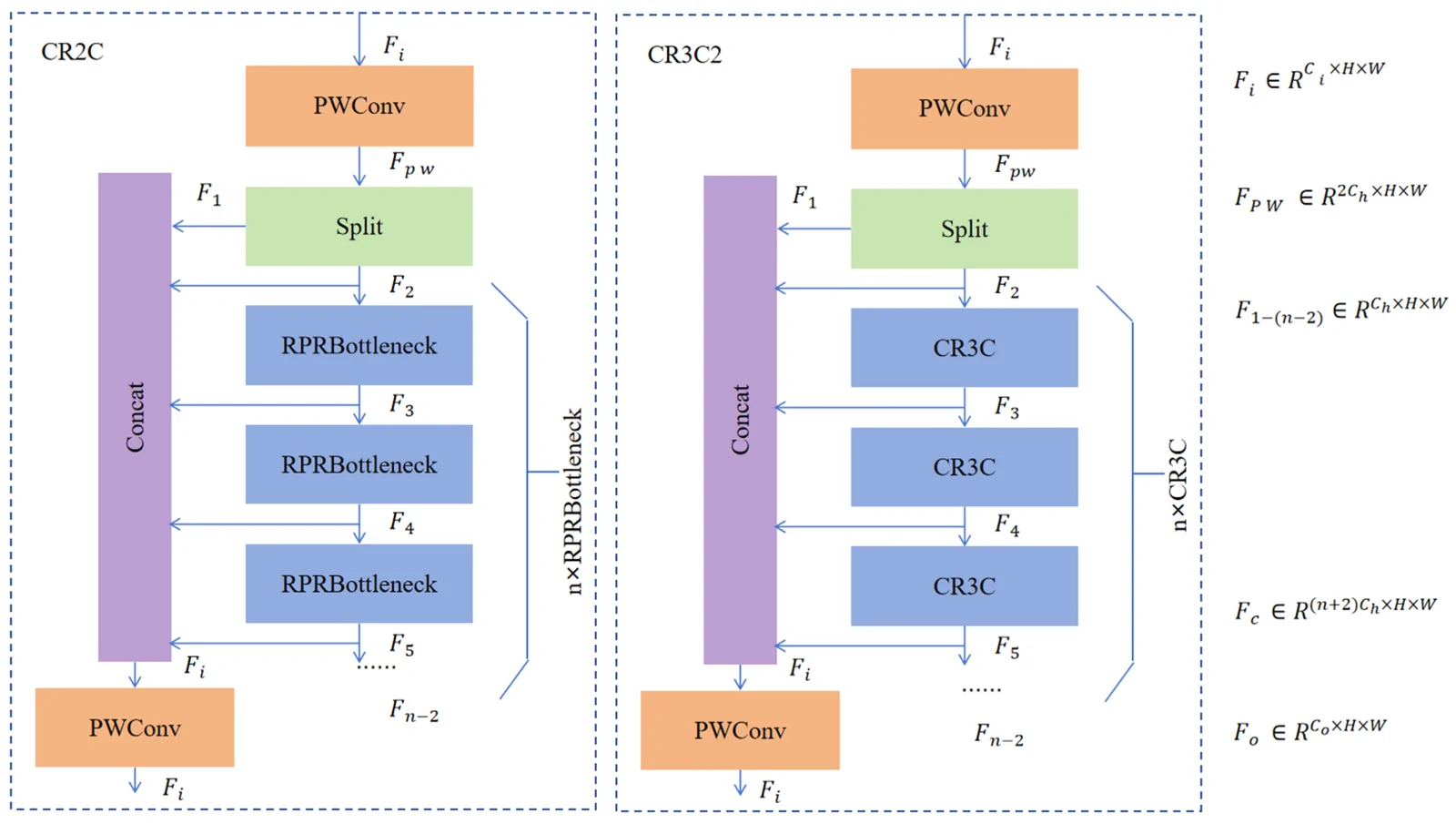
Architecture of the CR2C and CR3C2 blocks.

The CR2C and CR3C2 modules adopt the multi-branch feature fusion scheme of the CSP architecture, and their overall structure is illustrated in Figure 5. In this figure, $C_{i}$ denotes the number of input channels, $C_{o}$ denotes the number of output channels, $C_{h}=\left\lfloor e\cdot C_{o}\right\rfloor$ represents the number of hidden channels, and a stands for the stacking number of RPRBottleneck or CR3C.

The overall architectures of CR2C and CR3C2 are analogous. The input feature map *F_i_* first undergoes channel compression via the first pointwise convolution to yield *F*_1_, which is then equally split into two parts, *F*_2_ and *F*_3_ along the channel dimension. Herein, *F*_3_ serves as the input of the branch composed of stackable modules (RPRBottleneck or CR3C). Afterward, *F*_2_, *F*_3_ and the outputs of all stackable modules are concatenated along the channel dimension to generate *F_concat*. Finally, a second pointwise convolution is applied to restore the channel dimension, accomplishing multi-branch split-and-fuse feature fusion. This architecture effectively prevents the edge features of low-contrast defects from being attenuated during successive convolutions and significantly improves the model’s ability to recognize and characterize defect edge features. The corresponding computational procedures are given in (5)–(9), where *c*, *i*, and *j* denote the channel, row, and column indices of feature maps, respectively, and *k* represents the index of stackable modules.

(5) $$F_{pw}=PWConv(F_{i})$$

(6) $$\left\{\begin{array}{c} F_{1}(c,i,j)=F_{pw}(c,i,j)\\ F_{2}(c,i,j)=F_{pw}(c+C_{h},i,j) \end{array},c\in \left[1,C_{i}\right]\right.$$

(7) $$F_{k}=\frac{RPRBottleneck}{CR3C(F_{k-1})},k=2,3,\ldots ,n-2$$

(8) $$Fc=F_{1}⊕F_{2}⊕\cdots ⊕F_{n-2}$$

(9) $$F_{o}=PWConv(F_{c})$$

where *PWConv*(⋅) denotes pointwise convolution; *c*, *i*, and *j* represent the channel, row, and column indices of feature maps, respectively and *k* stands for the index of stackable modules; ⊕ denotes concatenation.

**Figure 6:**
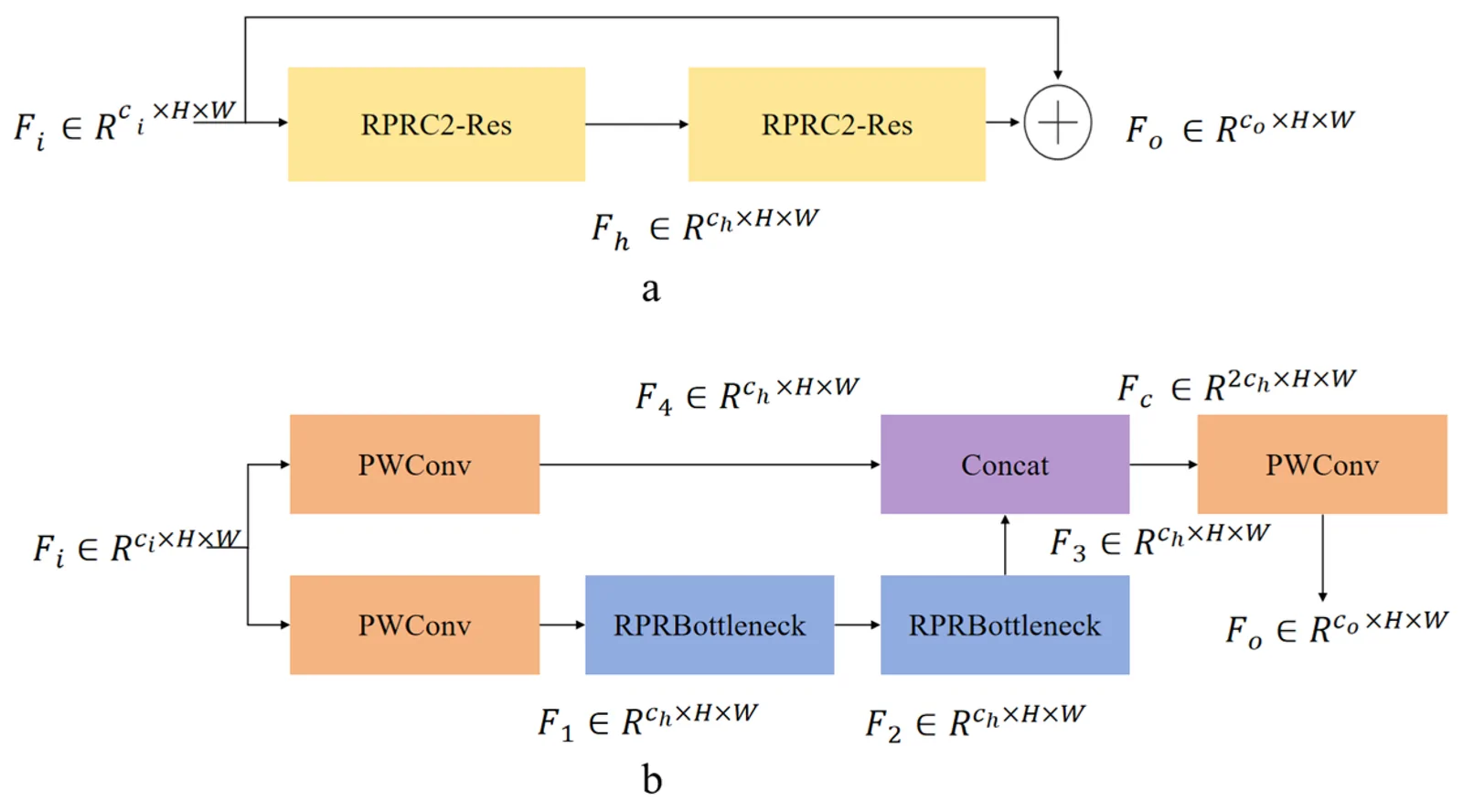
Architecture of Blocks: (**a**) RPRBottleneck; (**b**) CR3C.

There are distinct differences in parameter quantity and computational complexity between the CR2C and CR3C2 modules due to their different stacked unit architectures. The CR2C module adopts the RPRBottleneck as its stacked unit, which achieves lower computational complexity compared with the CR3C2 module that uses CR3C as the stacked unit. Accordingly, the CR2C module is mainly deployed in the shallow layers of the model for basic feature extraction while retaining detailed information contained in the original features, which can effectively avoid detail loss caused by excessive stacked convolutions. The detailed architecture of RPRBottleneck is shown in Figure 6a, where *C_i_* and *C_o_* represent the numbers of input and output feature map channels of this module, $C_{h}=\left\lfloor e\cdot C_{o}\right\rfloor$ denotes the number of hidden channels, and *e* is the scaling factor.

The computational procedure of the RPRBottleneck module is expressed in (10).

(10) $$F_{o}=RPRBottlenect(F_{i})=RPRC2-Res(RPRC2-Res(F_{i}))+F_{i}$$

By stacking multiple CR3C blocks, the CR3C2 module expands the number of branches and deepens the convolutional layers, endowing it with the capability to extract feature representations for defects across diverse scales. This architecture effectively tackles the significant inter-class discrepancies of hot-rolled strip surface defects and is tailored for high-level semantic feature extraction within the deep layers of the network. The detailed structure of the CR3C block is illustrated in Figure 6b.

The computational procedures of the CR3C block are formulated in (11)–(14).

(11) $$F_{3}=RPRBottleneck(RPRBottleneck(PWConv(F_{i})))$$

(12) $$F_{4}=PWConv(F_{i})$$

(13) $$F_{c}=F_{3}⊕F_{4}$$

(14) $$F_{o}=PWConv(F_{c})$$

### 3.4. TDAA: Two-Dimensional Axial Attention Module

The Two-Dimensional Axial Attention (TDAA) module efficiently captures long-range spatial dependencies by decomposing self-attention along height and width dimensions. Unlike standard self-attention with O(HW × HW) complexity, TDAA achieves O(HW × (H + W)) complexity, making it feasible for high-resolution industrial images.

Axial attention was originally proposed for medical image segmentation [23] and image generation, where 2D self-attention is decomposed into two sequential 1D operations along height and width axes. Our TDAA module adapts this axial decomposition principle to the defect detection task with two novel designs. First, it is strategically inserted at the C3 and C4 feature levels of the backbone instead of dense deployment, balancing global context modeling and computational cost. This placement proves especially effective for capturing the spatial context of defects without sacrificing real-time speed. Second, we combine axial attention with the reparameterized convolution backbone to build a hybrid architecture, where local low-contrast feature extraction and global context modeling complement each other. The axial attention concept itself is not new; its specific integration scheme for efficient surface defect detection constitutes the contribution of this work.

Axial Decomposition: The TDAA module processes attention sequentially along height and width dimensions. For an input feature map $X\in R^{C\times H\times W}$, we first apply height-wise axial attention:

(15) $$Y_{h}\left(i,j,:\right)=\sum_{k=1}^{H}soft\max\left(\frac{Q_{h}(i,j,:)\cdot K_{h}(k,j,:{)}^{T}}{\sqrt{d_{k}}}\right)V_{h}\left(k,j,:\right)$$

where $Q_{h},K_{h},V_{h}\in R^{d_{k}\times H\times W}$ represent query, key, and value projections for height-wise attention. For each column j, attention is computed across all rows k at that column position.

Following height-wise attention, width-wise axial attention is applied:

(16) $$Y_{w}\left(i,j,:\right)=\sum_{l=1}^{w}soft\max\left(\frac{Q_{w}(i,j,:)\cdot K_{w}(k,l,:{)}^{T}}{\sqrt{d_{k}}}\right)V_{w}\left(k,l,:\right)$$

The final output combines the original input with both attention branches through a gated fusion mechanism:

(17) $$Y=X+\sigma (W_{g}\cdot [Y_{h};Y_{w}])$$

where [⋅;⋅] denotes channel-wise concatenation, $W_{g}$ represents a 1 × 1 convolution for gating, and σ denotes sigmoid activation.

Computational Complexity Analysis: For an input of size H×W with C channels, standard self-attention requires $O(2C^{2}HW+2C(HW{)}^{2})$ operations. TDAA reduces this to $O(6C^{2}HW+2CHW(H+W))$ operations. For typical industrial inspection images with H=640,W=640,C=256, standard self-attention requires approximately $2C(HW{)}^{2}=2\times 256\times (640\times 640{)}^{2}\approx 8.6\times 10^{13}$ operations, while TDAA requires only $2CHW(H+W)=2\times 256\times 640\times 640\times (640+640)\approx 2.7\times 10^{11}$ operations, and the computational complexity was reduced by a factor of 320.

Defect-Specific Optimization: For surface defect detection, we incorporate relative positional encoding that explicitly models the spatial relationships between defect features. The relative positional encoding $R\in R^{(2H-1)\times d_{k}}$ is added to the attention scores:

(18) $$A\left(i,k,j\right)=\frac{Q_{h}(i,j,:)\cdot (K_{h}(k,j,:)+R(i-k+H){)}^{T}}{\sqrt{d_{k}}}$$

This positional encoding enables the model to better understand relative defect positions, which is critical for distinguishing between isolated defects and systematic manufacturing issues such as periodic roll marks.

### 3.5. MSF-FPN: Multi-Scale Feature Fusion Pyramid Network

Our MSF-FPN adopts bidirectional fusion principles and introduces two adaptations tailored to the extreme scale variation of surface defects: (i) adaptive feature weighting, in which each input feature map is assigned a learnable weight normalized by a softmax-like operation. This allows the network to dynamically balance semantic and detailed features according to defect scales; (ii) fusion optimization oriented toward small defects, where the P3 level receives extra contextual information from higher-resolution branches to boost detection of tiny defects such as micro-scratches. The bidirectional fusion idea is adapted from prior studies; the specific weighting mechanism and small-defect-oriented design are proposed in this work.

The Multi-Scale Feature Fusion Pyramid Network (MSF-FPN) extends the standard FPN architecture with adaptive feature weighting and cross-scale feature interaction. The network processes features at three levels (C3, C4, C5) from the backbone, with strides of 8, 16, and 32, respectively. Figure 2 shows the Neck block.

Adaptive Feature Weighting: During top-down feature fusion, each feature level receives adaptive weights based on its statistical characteristics. For each feature map $F_{l}$ at level l, we compute channel-wise and spatial-wise attention weights:

(19) $$\alpha_{l}=\sigma (AvgPool(F_{l})+MaxPool(F_{l}))$$

(20) $$\beta_{l}=\sigma (GAP(F_{l})\cdot W_{\beta })$$

where $\alpha_{l}\in R^{1\times H_{l}\times W_{l}}$ represents spatial attention weights, $\beta_{l}\in R^{C_{l}\times 1\times 1}$ represents channel attention weights, and $W_{\beta }$ is a learnable projection matrix. The weights are applied before feature fusion:

(21) $$F_{l}^{\prime }=\alpha_{l}⊗(\beta_{l}⊗F_{l})$$

where ⊗ denotes element-wise multiplication with broadcasting.

Bi-directional Feature Fusion: MSF-FPN incorporates both top-down semantic feature propagation and bottom-up detail feature enhancement. The top-down path fuses high-level semantic features with lower-level detailed features, while the bottom-up path propagates detailed spatial information back to higher levels. This bi-directional fusion ensures that each feature level contains both fine-grained details for small defect localization and high-level semantics for accurate defect classification. Scale-Adaptive Anchors: The FDetect detection head follows the anchor-based detection paradigm inherited from the YOLO series. Based on the statistical analysis of defect aspect ratios in hot-rolled strip steel datasets, we optimize the default anchor aspect ratios to [1:1, 1:3, 3:1], and adopt the Task-Aligned Assigner for target matching during training. The anchor sizes are dynamically adjusted during training based on input image resolution, ensuring consistent receptive field matching across different deployment scenarios.

### 3.6. FDetect: Cross-Branch Interactive Detection Head

**Figure 7:**
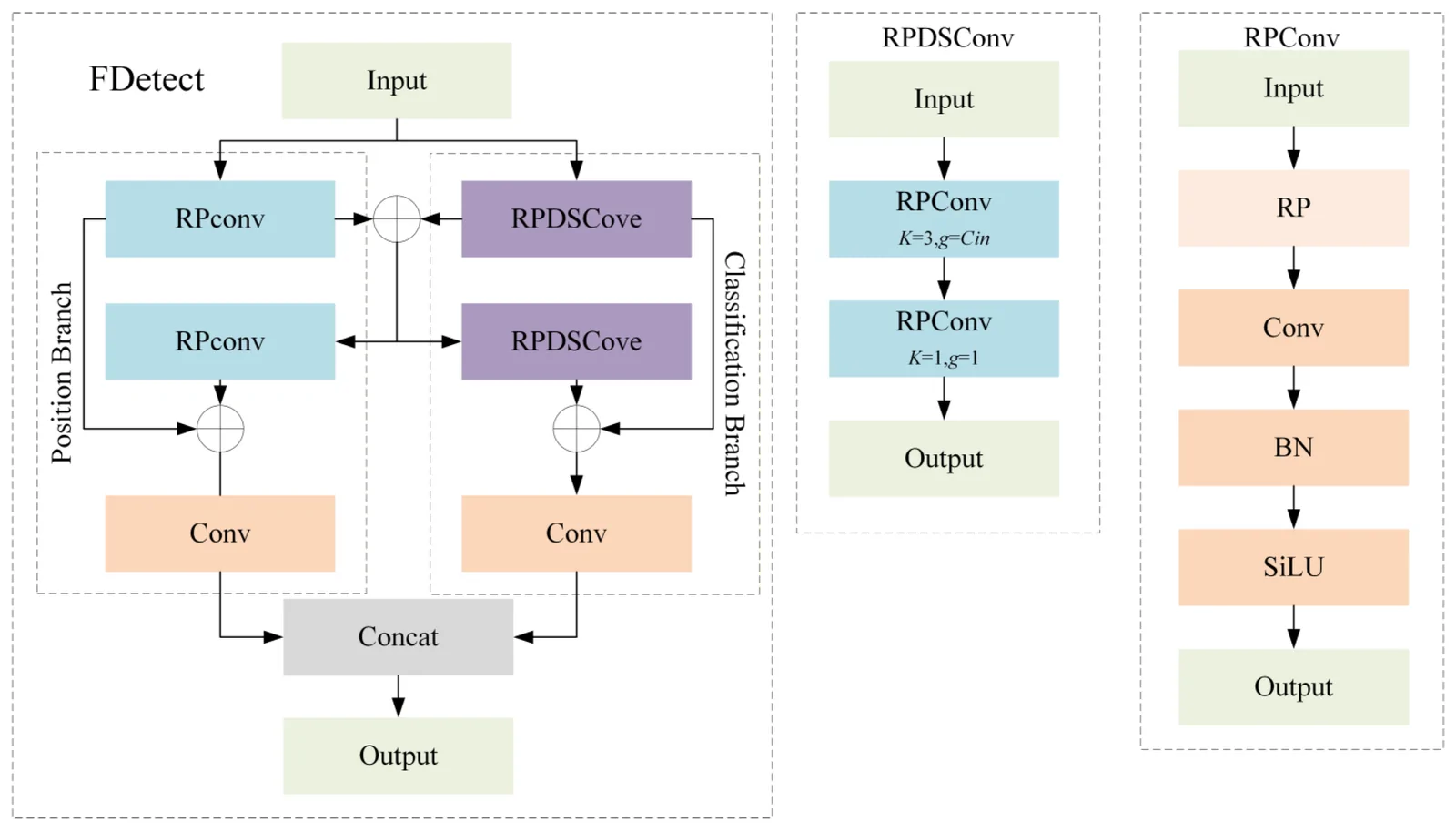
FDetect block Architecture Diagram.

The FDetect detection head employs a cross-branch interactive design that shares convolutional features between classification and regression branches while maintaining separate prediction layers. This design differs from the standard decoupled head approach, reducing parameter redundancy and improving feature utilization. The overall structure is illustrated in Figure 7.

The proposed FDetect head shares convolutional features between regression and classification branches while retaining separate prediction layers to cut redundant parameters. The regression branch stacks two RPConv blocks followed by a 1 × 1 convolution for precise defect localization; RPConv uses replication padding to preserve defect edge features. The classification branch adopts two lightweight RPDSConv blocks plus a 1 × 1 convolution to reduce computation cost, as classification primarily relies on high-level semantic features rather than fine-grained pixel details. After secondary feature mapping, each branch adds its initial feature, then the two outputs are concatenated for final detection prediction.

### 3.7. Adaptive Pruning Strategy

Our hybrid adaptive pruning strategy integrates BN scaling factors and feature-map rank analysis with a learnable weighting parameter λ. It automatically determines the per-layer pruning ratio according to the statistical properties of each layer instead of applying a uniform ratio across the whole network. The individual components (BN pruning and rank analysis) are well-established techniques; the hybrid adaptive framework and its application to lightweight defect detection models form the contribution of this work.

BN Scaling Factor Analysis: The scaling factor γ in Batch Normalization layers provides a natural measure of channel importance. Channels with small γ values contribute minimally to the output and can be safely pruned. We compute a channel importance score for each channel c:

(22) $$s_{c}^{bn}=\left|\gamma_{c}\right|$$

A global threshold $\tau_{bn}$ is determined such that pruning all channels with $S_{c}^{bn}<\tau_{bn}$ achieves the target parameter reduction ratio. The threshold is computed dynamically based on the distribution of scaling factors, ensuring stable pruning results across different training runs.

Feature Map Rank Analysis: Complementing BN scaling factors, we analyze the rank of feature maps to identify channels with low information content. For each layer, we compute the singular value decomposition (SVD) of output feature maps across a validation set:

(23) $$Fc=U_{C}\sum CV_{C}^{T}$$

The rank-based importance score is defined as the normalized cumulative energy of the first k singular values:

(24) $$s_{c}^{rank}=\frac{\sum_{i=1}^{k}\sigma_{c,i}}{\sum_{i=1}^{C}\sigma_{c,i}}$$

where $\sigma_{c,i}$ represents the i-th singular value of channel c. Channels with low rank scores produce low-rank feature maps containing redundant information.

Hybrid Pruning Decision: The final pruning decision combines both importance scores with adaptive weighting:

(25) $$s_{c}=\lambda \cdot \frac{s_{c}^{bn}}{max_{c}(s_{c}^{bn})}+\left(1-\lambda \right)\cdot s_{c}^{rank}$$

where λ controls the relative contribution of BN scaling factors and rank analysis. To systematically verify the optimal weighting of BN scaling factors and feature map rank analysis, we conduct a comprehensive sensitivity study of the hyperparameter *λ*, with values ranging from 0.1 to 0.9 in steps of 0.1. All experiments follow the same pruning pipeline: channel pruning based on the hybrid importance score, followed by 15-epoch fine-tuning with knowledge distillation. The quantitative results are demonstrated in Section 4.5.

As *λ* increases from 0.1 to 0.7, the parameter reduction rate and GFLOPs reduction rate rise steadily, while mAP50 remains stable above 75.5%. This indicates that BN scaling factors and feature map rank analysis play complementary roles: BN provides robust channel-level importance statistics, while rank analysis captures fine-grained information redundancy. The optimal accuracy-compression trade-off is achieved at *λ* = 0.7, yielding 36.3% parameter reduction and 49.0% GFLOPs reduction with only 0.5% mAP50 degradation. When *λ* exceeds 0.7, the strategy becomes overly dominated by BN scaling factors, leading to accelerated accuracy loss with only marginal gains in compression ratio. Therefore, *λ* = 0.7 is selected as the default setting for all pruning experiments in this work.

## 4. Experiments

### 4.1. Datasets

Two public steel surface defect datasets—NEU-DET and MVIT—are adopted to evaluate the proposed YOLO-RMF model. Detailed descriptions of each dataset are provided below.

(1) NEU-DET Dataset. The NEU surface defect dataset (NEU-DET) is a well-established benchmark collected by the Surface Defect Research Group at Northeastern University, China. It contains 1800 grayscale images at a fixed resolution of 200 × 200 pixels, covering six representative hot-rolled strip steel surface defects: crazing, inclusion, patches, pitted surface, rolled-in scale, and scratches. Each class contains exactly 300 samples, yielding a class-balanced distribution. The dataset is characterized by low-contrast defect appearances, similar morphologies across certain categories (e.g., rolled-in scale vs. pitted surface), and varied defect scales—all of which make it a challenging and representative benchmark for hot-rolled strip defect detection. We split the dataset into training, validation, and test sets in a 7:2:1 ratio (1260/360/180 images), preserving the balanced per-class distribution in all splits.
(2) MVIT Dataset. The MVIT metal surface defect dataset is collected from actual metal production lines and reflects more realistic factory imaging conditions. It contains 1200 high-resolution images with resolutions ranging from 96 × 106 to 3228 × 492 pixels, covering common defect patterns such as scratches, patches, and roll marks under complex lighting and background variations. Compared with NEU-DET, the MVIT dataset exhibits larger scale variation, more diverse defect morphologies, and more complex illumination, making it suitable for evaluating real-world generalization capability. The same 7:2:1 train/val/test split is applied.

Input Resolution and Preprocessing. During training and inference, all images are resized to a uniform resolution of 640 × 640 pixels using letterbox (aspect-ratio-preserving) resizing with bilinear interpolation, following standard practice in the YOLO detection framework. For the 200 × 200 NEU-DET images, this corresponds to an upsampling factor of approximately 3.2×. All GFLOPs and FPS values reported in this paper are measured at this 640 × 640 input resolution.

During training, we apply comprehensive data augmentation, including random horizontal/vertical flipping (*p* = 0.5), random rotation (−15° to +15°), random brightness adjustment (±30%), contrast enhancement (±20%), Gaussian noise injection (σ = 0.01), and Mosaic augmentation with four images. This augmentation strategy improves model robustness to the variations encountered in industrial production environments.

### 4.2. Evaluation Metrics

Detection performance is evaluated using standard COCO-style metrics:

mAP50: Mean Average Precision at Intersection over Union (IoU) threshold 0.50
mAP50-95: Mean Average Precision averaged over IoU thresholds from 0.50 to 0.95 in increments of 0.05
Precision: Ratio of true positives to total detections
Recall: Ratio of true positives to total ground truth objects
FPS: Frames Per Second, measured with batch size 1 on a single GPU

Model complexity metrics include the following:

Parameters: Total number of trainable parameters
GFLOPs: Billion floating-point operations for single image inference at 640 × 640 resolution
Memory Usage: Peak GPU memory consumption during inference
Speed evaluation protocol: All GPU inference speeds (FPS) are measured on a single NVIDIA RTX 4090 GPU with batch size 1 under FP32 precision. Results are averaged over 100 warm-up runs and 1000 test iterations, including post-processing (non-maximum suppression, NMS). Jetson AGX Orin speeds are obtained via TensorRT 8.5 under the standard 25 W balanced power mode with batch size 1, and reported for both FP16 and INT8 precision. The end-to-end system FPS (Table 1) further incorporates latency from image capture, preprocessing and post-processing. All FPS values in the manuscript follow these protocols.

### 4.3. Implementation Details

All experiments are implemented in PyTorch 2.1 with CUDA 12.1 and cuDNN 8.9. Training is performed on a single NVIDIA RTX 4090 GPU with 24 GB of memory. The model is trained for 1000 epochs with a batch size of 16 using stochastic gradient descent with momentum 0.937 and weight decay 0.0005. The initial learning rate is 0.01, reduced by a factor of 10 at epochs 200 and 260, respectively, with cosine annealing applied throughout the training process. Input image resolution is 640 × 640 pixels for all experiments. Multi-scale training with random resolution between 480 and 800 pixels is applied for the first 200 epochs, followed by fixed 640 × 640 resolution for the remaining epochs. For the pruning experiments, we set a pruning ratio search range from 20% to 50%, with 40% as the initial reference target for compression intensity. All pruned models are fine-tuned for 15 epochs at a learning rate of 0.001 with knowledge distillation. The optimal pruning configuration is determined via an ablation study and adopted for subsequent edge deployment verification. To ensure fair comparison, all experiments adopt a fixed random seed of 0 for data shuffling, weight initialization, and augmentation sampling. The same seed protocol and training pipeline are applied to every compared baseline. The following weights are used: 7.5 for box regression loss (CIoU), 1.0 for classification loss (BCE), and 1.5 for distribution focal loss (DFL). During inference, the confidence threshold is set to 0.001, and the NMS IoU threshold is set to 0.7 for NEU-DET and 0.6 for MVIT. The Task-Aligned Assigner uses topk = 10, α = 0.5, and β = 6.0 as default hyperparameters. Deployment testing is conducted on NVIDIA Jetson AGX Orin with 32GB memory, running JetPack 5.1. TensorRT 8.5 is used for model optimization with half-precision floating-point (FP16) and 8-bit integer (INT8) quantization.

### 4.4. Overall Performance Comparison

**Figure 8:**
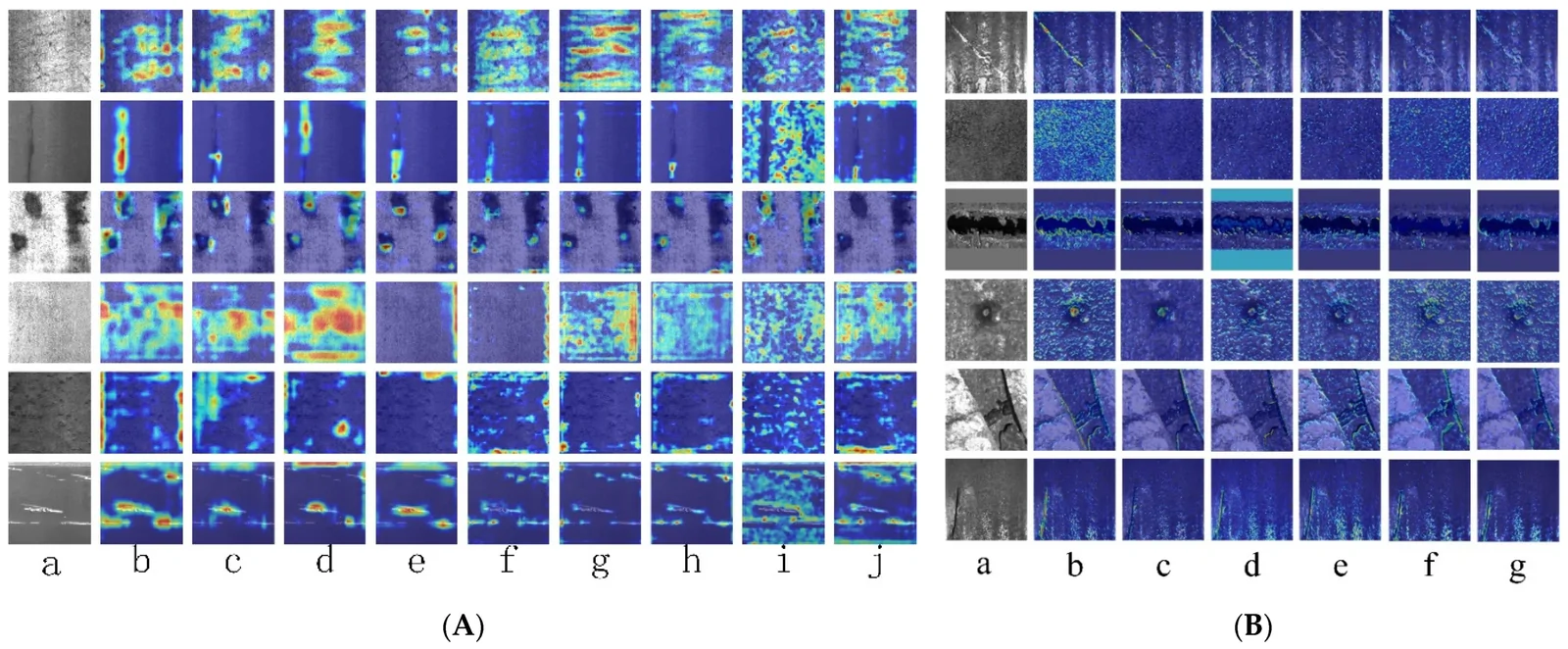
Visual comparison of detection results. (**A**) Results on NEU-DET. From top to bottom: crack, inclusion, patch, pitting, rolled-in scale, scratch. (**a**) Original; (**b**) YOLO-RMF; (**c**) Steel-DETR; (**d**) Defect-YOLO; (**e**) YOLOv8s; (**f**) YOLOv8n; (**g**) YOLOv7-tiny; (**h**) YOLOv5s; (**i**) RetinaNet; (**j**) Faster R-CNN. (**B**) Results on MVIT. (**a**) Original; (**b**) YOLO-RMF; (**c**) YOLOv8; (**d**) YOLOv8n; (**e**) YOLOv7-tiny; (**f**) YOLOv5s; (**g**) Faster R-CNN.

Table 1 lists quantitative results on NEU-DET. YOLO-RMF achieves the highest mAP50 (76.1%) and mAP50-95 (40.6%) with 212 FPS inference speed. Compared with YOLOv8s, it gains 1.6% mAP50 and 1.2% mAP50-95, cuts GFLOPs by 25.2% and lifts FPS by 32.5%. Visual detection contrast in Figure 8A demonstrates that YOLO-RMF better identifies subtle low-contrast defects missed by other detectors. On the mAP50 versus mAP50-95 gap. The notable discrepancy between mAP50 (76.1%) and mAP50-95 (40.6%) is a typical trait of surface defect detection benchmarks and requires explicit interpretation. Unlike common objects with sharp, well-defined boundaries, many steel surface defects, including crazing, rolled-in scale and diffuse patches, have irregular shapes, gradual intensity variations and blurred edges. As the IoU threshold rises from 0.50 to 0.95, these indistinct boundaries trigger a rapid decline in matching rates and yield a large gap between mAP50 and mAP50-95. The 200 × 200 resolution of NEU-DET further constrains boundary localization precision under high IoU conditions.

From a practical perspective, mAP50 characterizes detection sensitivity, i.e., whether defects can be correctly identified, which generally serves as the primary quality gate for industrial inspection. By contrast, mAP50-95 represents localization precision, which is more vital for downstream tasks, including automatic size measurement and defect severity grading. Our model achieves excellent performance on both metrics among lightweight detectors, demonstrating simultaneous improvements in detection and localization capabilities.

On the more complex MVIT dataset (Table 2), YOLO-RMF still achieves 72.4% mAP50 and 38.2% mAP50-95, outperforming all lightweight and two-stage baselines. Figure 8B visualization verifies its stronger adaptability to real factory high-resolution images.

### 4.5. Ablation Studies

Ablation studies are conducted on NEU-DET to verify the effectiveness of each proposed component. Starting from a baseline YOLOv8n architecture, we sequentially (cumulatively) integrate each proposed module, so that every configuration is obtained by adding one new module to the immediately preceding configuration. Consequently, the gains reported in Table 3a represent the incremental improvement over the preceding configuration, rather than the isolated contribution of each module in the absence of the others.

The ablation results of Table 3a confirm that each proposed component provides a consistent accuracy gain when added to the preceding configuration: integrating RPRC2 improves mAP50 by 1.25% over the baseline with no increase in computational cost; adding CR2C/CR3C2 further increases mAP50 by 0.51%; adding TDAA yields an additional 0.53% mAP50 gain with manageable computational overhead and MSF-FPN and FDetect Head provide further incremental improvements of 0.33% and 0.32% in mAP50, respectively. At the same time, we also conduct separate tests for each module. Table 3b shows the contribution of each module to model performance when acting independently.

Table 4a shows the quantitative results from the sensitivity study on hyperparameter λ, and Table 4b demonstrates the impacts of varying pruning ratios on model performance.The ablation results show that the set pruning ratio of 35% achieves the optimal accuracy-compression trade-off. It corresponds to an actual parameter reduction of 36.3% and an actual GFLOPs reduction of 49.0%, with only 0.5% mAP50 degradation and 19.8% inference speed improvement. Pruning ratios above 35% yield marginal additional compression but cause increasingly severe accuracy loss. This optimal 35% pruning configuration is used for all subsequent deployment experiments on the Jetson AGX Orin platform.

### 4.6. Per-Class Performance Analysis

**Figure 9:**
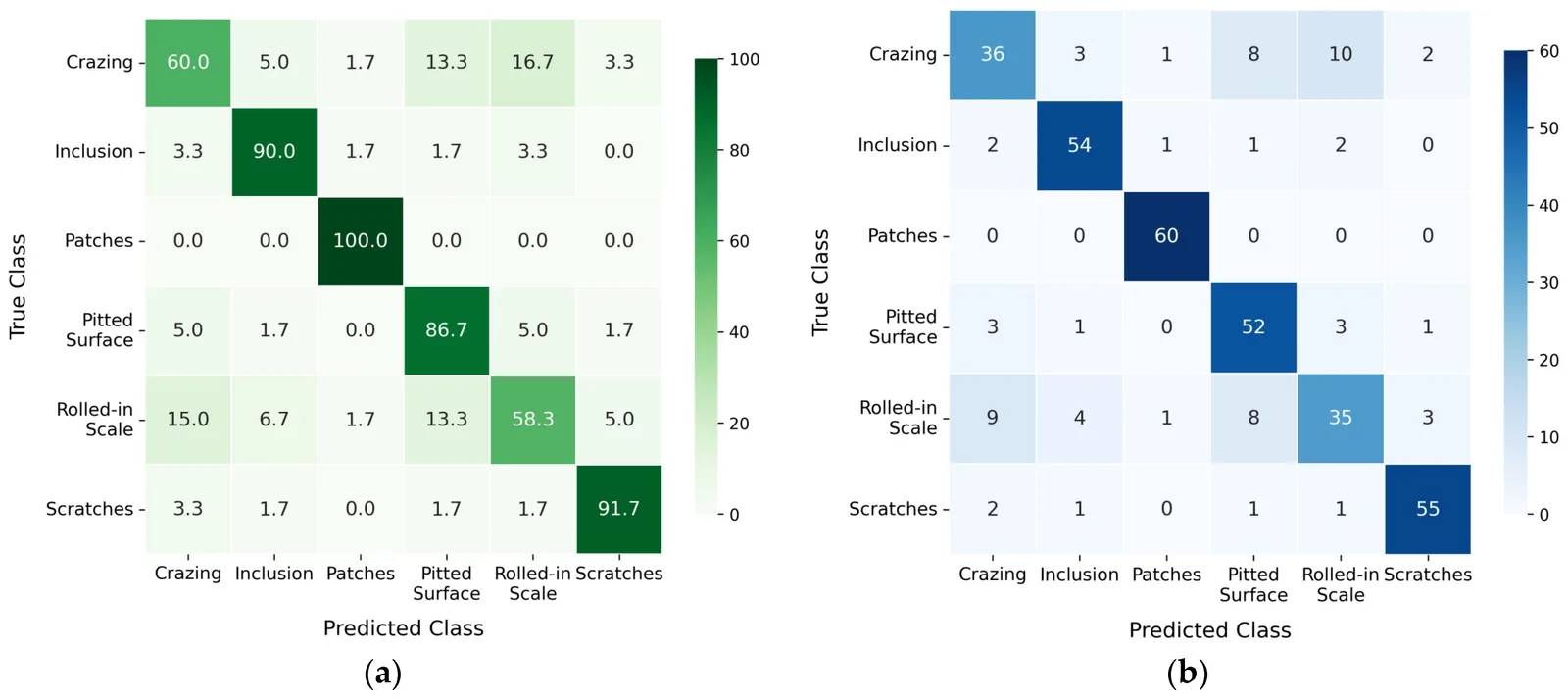
(**a**). YOLO-RMF Confusion Matrix (Row-Normalized, %) on NEU-DET. (**b**). YOLO-RMF Confusion Matrix (Absolute) on NEU-DET.

A question of practical importance is whether YOLO-RMF can reliably detect various types of surface defects, given the large visual differences across defect categories. To address this, we present per-class detection results on the NEU-DET dataset in this section, complemented by a confusion matrix analysis. The analysis results are refer to Figure 9a,b. The results confirm that YOLO-RMF successfully identifies all six defect types, with performance varying according to the inherent visual difficulty of each category. Table 5 presents per-class detection performance on NEU-DET, providing insights into the model’s strengths across different defect types.

Table 5 presents per-class detection performance on the NEU-DET dataset, which intuitively reflects the model’s performance across different defect types. The model achieves the best results for patches (AP50 = 93.5%, F1 = 0.984), followed by scratches (AP50 = 85.9%, F1 = 0.905). These two defect categories are relatively large, highly distinctive, and possess clear boundaries. In contrast, rolled-in scale (AP50 = 55.0%, Recall = 0.586) and crazing (AP50 = 56.4%, Recall = 0.601) yield the lowest detection performance. These two defects exhibit subtle appearances, low contrast and highly irregular shapes, making them difficult to distinguish from normal surface texture. The same trend applies to mAP50-95: patches achieve the highest value (49.5%) while rolled-in scale obtains the lowest (30.4%). Nevertheless, classes with ambiguous boundaries (crazing and rolled-in scale) suffer a greater relative performance drop from mAP50 to mAP50-95, verifying that boundary fuzziness dominates the localization accuracy gap.

The normalized confusion matrix (Figure 9) reveals two major misclassification patterns. First, rolled-in scale is frequently misclassified as crazing and pitted surface (approximately 41.7% of rolled-in scale classification errors). This is because the three categories have overlapping gray-level distributions and share fragmented, scale-like textures. Second, crazing suffers from substantial missed detections (Recall = 0.601), consistent with its extremely fine crack patterns that resemble image noise. By comparison, patches show nearly no classification confusion (Recall = 0.997) due to their large, compact, high-contrast regions.

False positive analysis. Most false positives originate from background textures that resemble defects. For instance, oxide-scale speckles on steel surfaces are occasionally detected as crazing or pitted surfaces. Such background-induced false positives are more prominent in the two lowest-performing classes, which accounts for their lower precision.

False negative analysis. False negatives are dominated by low-contrast, small-area defects, especially crazing and rolled-in scale, whose boundaries are heavily blurred by surface noise. Features enhanced by TDAA attain higher recall and mitigate this issue to some extent; nevertheless, residual missed detections remain. This indicates an inherent difficulty in detecting extremely subtle defects under the 200 × 200 resolution.

### 4.7. Cross-Dataset Generalization

To evaluate cross-dataset generalization capability, we train models on NEU-DET and test directly on MVIT without fine-tuning. The comparison results are shown in Table 6.

YOLO-RMF achieves better cross-dataset generalization, with a relative performance drop of only 37.8%, compared with 40.5–46.4% for other competing methods (Table 6). This finding shows that the full YOLO-RMF model preserves detection performance more effectively when deployed on a new dataset without fine-tuning. We hypothesize that the axial-attention-based global context modeling in TDAA and the reparameterized convolution in RPRC2 facilitate such improved transferability by learning more domain-invariant feature representations. Nevertheless, separating the individual contribution of each component to cross-dataset generalization demands extra ablation experiments, which we mark as future work.

### 4.8. Robustness Testing

Industrial inspection systems must operate reliably under various environmental disturbances. We evaluate robustness under five types of common perturbations: Gaussian noise, salt-and-pepper noise, brightness variation, contrast reduction, and blur. We note that several of these perturbations (Gaussian noise, brightness, and contrast) conceptually overlap with data augmentation adopted in training. Nevertheless, the perturbation magnitudes tested herein are considerably larger than those used for augmentation (e.g., brightness of −40% versus ±30% during training; Gaussian noise with σ = 0.10 versus σ = 0.01 during training). Hence, these results mainly reflect robustness beyond the range of training augmentation, rather than robustness to variations already encountered in training. In contrast, salt-and-pepper noise and Gaussian blur are not applied in training and correspond to unseen perturbations. There are two severity levels for Gaussian noise, resulting in six experimental settings in total. The comparison results are shown in Table 7.

YOLO-RMF consistently surpasses YOLOv8n under all perturbation types, with relative gains ranging from 5.6% to 9.2% (Table 7). The most prominent improvements are obtained on unseen perturbations, including salt-and-pepper noise (+6.3%) and Gaussian blur (+6.0%), as well as out-of-range brightness variation (+8.2%), demonstrating genuine robustness to conditions not covered by training augmentation. Such enhanced robustness may stem from the combined effects of multi-branch training within RPRC2 modules and global context modeling in TDAA, which yield more stable feature representations under adverse conditions. We acknowledge that our robustness comparison is currently limited to YOLOv8n as the baseline. Additional comparisons with other detectors will reinforce this analysis and are reserved for future work.

### 4.9. Deployment Verification

To systematically verify the real-time capability of YOLO-RMF for hot-rolled strip steel inspection, we evaluate the inference speed at three levels: GPU inference, edge deployment, and end-to-end system latency. The results are compared against the minimum frame rate requirement of 200 FPS derived in Section 1.1 (based on a line speed of 25 m/s and a 125 mm FOV). Deployment performance is evaluated on NVIDIA Jetson AGX Orin, a representative edge computing platform for industrial applications. The Jetson AGX Orin is configured in its standard 25 W balanced power mode for edge deployment, as specified in the manufacturer’s official product datasheet. This power mode delivers a balanced trade-off between inference throughput and power consumption. The comparison results are shown in Table 8.

Notably, the unpruned YOLO-RMF achieves lower FPS under INT8 quantization (126 FPS) than FP16 (147 FPS) on Jetson AGX Orin. This counterintuitive observation originates from two primary factors. First, the TDAA axial attention module and RPRC2 reparameterized convolutions contain operations that are not fully optimized for INT8 in TensorRT (e.g., softmax, element-wise operations and custom padding). This forces some layers to fall back to FP16 and introduces repeated type-conversion overhead between INT8 and FP16 computation paths. Second, for lightweight models such as YOLO-RMF, FP16 inference is already memory-bandwidth limited on Jetson AGX Orin. Under batch size 1, latency is dominated by memory access instead of arithmetic throughput, so the theoretical computational benefit of INT8 cannot be fully exploited. After pruning, the model becomes more compact with regularized structures, reducing fallback operations and enabling obvious speedup with INT8.

The pruned YOLO-RMF model reaches 169 FPS under FP16 precision and 233 FPS with INT8 quantization on Jetson AGX Orin. According to the frame-rate calculation presented below, this throughput can theoretically support line speeds up to approximately 29 m/s under the assumed 160 mm FOV configuration, demonstrating that the algorithm possesses sufficient computational headroom for high-speed hot-rolling inspection. The memory footprint of 218 MB with INT8 quantization enables deployment on resource-constrained edge devices with limited memory.

The total end-to-end latency of 6.3 ms corresponds to an effective detection frame rate of ~158.7 FPS. With a strip running speed of 25 m/s (25,000 mm/s), the distance the strip travels between two consecutive frames is as follows:

(26) $$\Delta x_{step}=\frac{v}{FPS}=\frac{25000\;m/s}{158.7}\approx 158\;\mathrm{mm}\;per\;frame$$

With a 160 mm field-of-view (FOV) along the rolling direction, adjacent frames maintain full overlap coverage and ensure gap-free inspection. For a typical industrial deployment configuration with 1280 effective pixels along the rolling direction, the per-pixel spatial resolution is as follows:

(27) $$\Delta x_{pixel}=\frac{FOV}{pixel\;count}=\frac{160}{1280}=0.125\;\mathrm{mm}\;per\;pixel$$

This gives a theoretical per-pixel spatial resolution of ~0.125 mm under the assumed 160 mm FOV and 1280 effective pixels along the rolling direction, corresponding to sub-millimeter resolution. We stress that this value relies on the specific camera sensor, lens and working distance in practical deployment and is provided herein as a theoretical reference instead of an on-site measured specification.

Table 9 indicates the complete inspection system achieves end-to-end latency of 6.3 ms, corresponding to over 158 samples per second—sufficient for inspecting hot-rolled strip moving at 25 m/s with sub-millimeter spatial resolution.

### 4.10. Statistical Significance Test

To eliminate random training fluctuation interference, each ablation configuration and comparison baseline is trained 10 times with independent random seeds, and mAP metrics are analyzed statistically.

**Figure 10:**
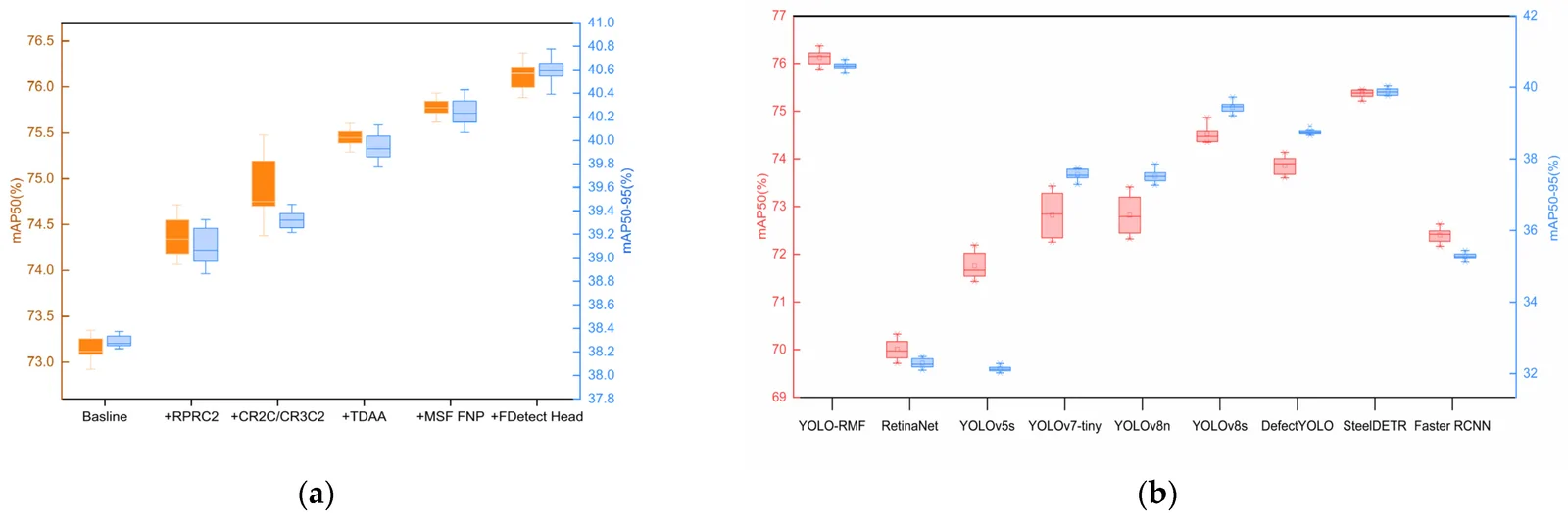
Visualization of Statistical significance test. (**a**) A dual-Y box plot visualizes mAP50 and mAP50-95 distributions from ten repeated training trials. With sequential integration of RPRC2, CR2C/CR3C2, TDAA, MSF-FPN and FDetect Head, the mean and median of both metrics rise monotonically. Low sample dispersion across all groups verifies stable model performance. (**b**) Dual-axis box plots illustrate mAP50 (red, left Y-axis) and mAP50-95 (blue, right Y-axis) of all detectors, each trained 10 times with unique random seeds.

For the ablation groups, the Shapiro–Wilk test confirms normality (all *p* > 0.05), while Levene’s test indicates a mild violation of the homogeneity-of-variances assumption (*p* = 0.017). Since equal variances could not be assumed, we employed Welch’s one-way ANOVA, which does not require homogeneous variances, in place of the standard ANOVA. Welch’s ANOVA revealed a highly significant overall difference among the six configurations (Welch’s F(5, 24.78) = 546.77, *p* < 0.001). Pairwise comparisons were then performed using the Games-Howell post-hoc test, which likewise relaxes the equal-variance assumption; all pairwise differences were significant (adjusted *p* < 0.01), confirming that the incremental accuracy gains obtained by sequentially adding RPRC2, CR2C/CR3C2, TDAA, MSF-FPN, and FDetect are statistically robust. We additionally report 95% confidence intervals and effect sizes (Cohen’s d) in Table 10. Figure 10a and Table 10 show monotonic growth of mAP50 and mAP50-95 with low result dispersion, proving stable performance improvement of each module.

To verify YOLO-RMF’s superiority, we train YOLOv5s, RetinaNet and other baselines 10 times with separate random seeds and extract converged mAP50/mAP50-95 for statistical analysis. Because the Shapiro–Wilk test shows that some baselines violate normality, we adopted the distribution-free Kruskal–Wallis test as an omnibus test for overall inter-group differences, followed by Dunn’s test with Bonferroni correction for pairwise comparisons to control the family-wise error rate. The Kruskal–Wallis test returned a significant overall difference among all nine detectors for both mAP50 (H = 85.53, *p* < 0.001) and mAP50-95 (H = 86.84, *p* < 0.001). Dunn’s test showed that YOLO-RMF significantly outperformed five of the eight baselines (RetinaNet, YOLOv5s, YOLOv7-tiny, YOLOv8n, and Faster R-CNN) at the Bonferroni-adjusted *p* < 0.01 level on mAP50. For the three strongest baselines (YOLOv8s, Defect-YOLO, and Steel-DETR), the rank differences (20, 30, and 10, respectively) did not reach the adjusted significance threshold, which is consistent with their closer performance to YOLO-RMF (Table 11). All 10 repeated mAP results of YOLO-RMF surpass those of baselines, fully verifying the validity of our improvements. Comparative results are visualized in Figure 10b and listed in Table 11.

## 5. Discussion

### 5.1. Speed-Accuracy Trade-Off Analysis

The experimental results demonstrate that YOLO-RMF achieves an improved speed-accuracy trade-off for surface defect detection. The key insight enabling this performance improvement is the combination of structural reparameterization and decomposed attention mechanisms.

Unlike traditional approaches that improve accuracy at the cost of speed or vice versa, the RPRC2 module enhances feature extraction capability exclusively during training without imposing any inference-time overhead. This design achieves a favorable balance between the two: the representational power of multi-branch architectures during training and the efficiency of single-path architectures during deployment.

The TDAA module further demonstrates that global context modeling can be achieved efficiently through axial decomposition. While Standard full self-attention would introduce prohibitive computational overhead for high-resolution industrial images, making it impractical for real-time deployment, TDAA provides a 0.56% mAP50 improvement with only a 1.37% FPS reduction. This efficiency is critical for industrial applications where each millisecond of latency directly translates to production throughput. Beyond inference speed and detection accuracy, our model achieves improved localization precision. It obtains an mAP50-95 of 40.6%, 0.6 percentage points higher than Steel-DETR (39.8%). This benefit is valuable for applications such as automated defect size classification, which demands precise boundary localization.

### 5.2. Industrial Application of YOLO-RMF

This section describes the industrial applicability and edge deployment framework of YOLO-RMF based on the deployment experimental results presented in Section 4.9. The deployment results on Jetson AGX Orin indicate that YOLO-RMF is capable of meeting the typical requirements of real-world industrial edge deployment. The pruned model with INT8 quantization achieves 233 FPS with a 218 MB memory footprint, enabling the following practical benefits:

1. Edge Deployment Feasibility: With 218 MB memory consumption, the model fits well within the memory limits of compact industrial edge controllers (e.g., Jetson AGX Orin-class devices). Such hardware costs less than industrial GPU servers and reduces the total hardware investment of the system.
2. Power Efficiency: Jetson AGX Orin consumes only 25W during operation, compared with 450W for a desktop GPU, significantly reducing operational energy costs for 24/7 continuous operation.
3. Robustness: The demonstrated robustness to out-of-range noise, illumination variations, and blur (Section 4.8) suggests promising performance under challenging imaging conditions. Nevertheless, full validation within real production environments with extra disturbance types such as steam and vibration remains future work.

The above results demonstrate the practical deployability of YOLO-RMF (accuracy, speed and memory footprint on Jetson AGX Orin). Nevertheless, full industrial deployment additionally requires (i) optical system design (camera selection, lighting and lenses), (ii) mechanical integration (IP-rated enclosure, vibration isolation and heat dissipation), (iii) long-term field reliability tests (thermal drift, lens contamination and seasonal variations), and (iv) integration with production-line control systems (PLC, MES). These topics lie outside the scope of the present algorithm-oriented work and will be pursued in future industrial collaborations.

### 5.3. Limitation Regarding Class Imbalance

As described in Section 4.1, both NEU-DET and MVIT are approximately class-balanced benchmarks (NEU-DET: 300 images per class). This differs from real hot-rolling production lines, where defect classes exhibit a long-tailed distribution—for example, scratches and rolled-in scale occur frequently, while holes and inclusions are rare. The balanced evaluation setting has two implications for our results. First, the reported mAP values reflect average performance over equally weighted classes, and thus may overestimate the model’s practical detection ability for rare defect types, which contribute little to the mean when classes are balanced. Second, training on a balanced dataset does not exercise the model’s ability to handle skewed gradients or dominated background classes, which are common failure modes under severe imbalance in industry. To mitigate this gap in future work, we plan to (i) evaluate on imbalanced industrial datasets collected from real production lines, and (ii) incorporate class-imbalance-aware training strategies such as re-sampling and cost-sensitive loss functions (e.g., Focal Loss). We believe the balanced benchmarks still provide a fair and reproducible basis for comparing detection architectures; however, the class-imbalance limitation should be kept in mind when extrapolating our quantitative results to production scenarios.

## 6. Conclusions

This paper proposes YOLO-RMF, a real-time one-stage detector for hot-rolled strip steel surface defect detection, with four core innovations. The reparameterized 2 × 2 convolution (RPRC2) enhances low-contrast feature extraction while reducing computational cost. The two-dimensional axial attention (TDAA) implements long-range spatial modeling with linear complexity. The adaptive-weighted multi-scale feature fusion pyramid network (MSF-FPN) balances fine details of small defects and semantic information of large defects. A hybrid adaptive pruning strategy combining BN scaling factors and feature-map rank analysis is adopted for model compression.

Experiments on the NEU-DET and MVIT datasets verify the effectiveness of YOLO-RMF. On NEU-DET, the model achieves 76.1% mAP50 and 40.6% mAP50-95 at 212 FPS on RTX 4090 and can identify all six types of defects. The cross-dataset test on MVIT reaches 72.4% mAP50, demonstrating favorable generalization performance. The hybrid pruning reduces model parameters by 36.3% and GFLOPs by 49.0%, with only a 0.5% drop in mAP50.

YOLO-RMF meets the real-time requirement for high-speed hot-rolling inspection. It runs at 212 FPS on RTX 4090 and 233 FPS with INT8 quantization on Jetson AGX Orin, with an end-to-end system latency of 6.3 ms. These performances satisfy the minimum 200 FPS corresponding to a strip speed of 25 m/s and a 125 mm field of view, making it suitable for edge-computing-based industrial inspection. All hyperparameters are provided in this paper, and source code is available upon reasonable request.

Limitations remain in this work. TDAA introduces extra computational overhead. Evaluations are currently conducted only on public datasets; on-site tests on real production lines are required to assess the impacts of steam, vibration and thermal drift on long-term reliability. Public datasets have balanced class distributions and cannot represent the long-tailed defect distribution in industrial scenarios. Future work will investigate sparse axial attention, more powerful structured pruning, continuous learning on production streaming data, and on-site deployment and va lidation on hot-rolling lines in cooperation with industrial enterprises.

## Figures and Tables

**Table 1:** Performance comparison on NEU-DET dataset.

Method	Backbone	mAP50 (%)	mAP50-95 (%)	Parameters (M)	GFLOPs (B)	FPS
**YOLO-RMF**	**CSP-RPRC2**	**76.1**	**40.6**	**8.6**	**21.4**	**212**
RetinaNet	ResNet-50	69.8	32.1	36.5	98.2	28.7
YOLOv5s	CSPDarknet	71.5	36.8	7.2	16.5	142
YOLOv7-tiny	ELAN	72.8	37.5	6.2	13.8	168
YOLOv8n	CSPDarknet	73.1	37.8	3.2	8.7	225
YOLOv8s	CSPDarknet	74.5	39.4	11.2	28.6	160
Defect-YOLO	Modified CSP	73.8	38.7	9.8	24.3	125
Steel-DETR	ResNet-50	75.2	39.8	45.6	156.2	18.5
Faster R-CNN	ResNet-50	72.3	35.2	41.2	128.5	15.3

Note: All detection metrics are reported as the mean of 10 independent training runs with different random seeds. Inference speed (FPS) and complexity metrics are measured on a single NVIDIA RTX 4090 GPU with batch size 1 and 640 × 640 input resolution (after letterbox resizing from the native dataset resolution).

**Table 2:** Performance comparison on MVIT dataset.

Method	mAP50 (%)	mAP50-95 (%)	Parameters (M)	GFLOPs	FPS
YOLO-RMF	72.4	38.2	2.15	6.5	212
YOLOv8	68.1	35.2	2.56	6.3	158
YOLOv8n	65.9	33.8	2.6	6.4	212
YOLOv7-tiny	64.4	33.6	2.27	6.5	181
YOLOv5s	61.3	31.8	30.5	86.1	22
Faster R-CNN	60.6	30.9	41.32	38.9	46

**Table 3:** (**a**) Ablation study of proposed modules. (**b**) The effect of each improvement module on the baseline model.

(**a**)											
**Configuration**				**mAP50 (%)**			**mAP50-95 (%)**		**GFLOPs**		**FPS**
Baseline (YOLOv8n)				73.13			38.2		8.7		225
+RPRC2				74.38			39.09		8.7		225
+CR2C/CR3C2				74.89			39.30		12.4		218
+TDAA				75.45			39.94		16.8		215
+MSF-FPN				75.78			40.23		19.2		213
**+FDetect Head**				**76.10**			**40.60**		**21.4**		**212**
Full Model + Pruning				75.6			40.1		10.9		254
(**b**)											
**Code**	**RPRC2**	**CR2C/CR3C2**	**MSF-FPN**		**TDAA**	**FDetect Head**		**FPS**	**mAP50 (%)**	**mAP50-95 (%)**	
1	-	-	-		-	-		225	73.13	38.2	
2	√	-	-		-	-		225	74.38	39.09	
3	-	√	-		-	-		212	73.9	39.0	
4	-	-	√		-	-		212	73.53	38.8	
5	-	-	-		√	-		215	73.65	38.8	
6	-	-	-		-	√		212	73.73	38.9	

**Table 4:** (**a**) Sensitivity analysis of the hybrid weighting parameter λ. (**b**) Pruning ratio ablation.

(**a**)								
**λ**	**mAP50 (%)**		**mAP50-95 (%)**		**Param. Reduction**		**GFLOPs Reduction**	
0.1	75.7		40.2		21.8%		31.9%	
0.2	75.7		40.1		25.1%		36.2%	
0.3	75.6		40.0		28.7%		41.3%	
0.4	75.6		39.9		31.2%		44.0%	
0.5	75.5		39.9		33.5%		46.3%	
0.6	75.6		40.0		35.1%		47.8%	
**0.7**	**75.6**		**40.1**		**36.3%**		**49.0%**	
0.8	75.2		39.7		38.9%		51.5%	
0.9	74.7		39.2		41.5%		54.2%	
(**b**)								
**Pruning Ratio**		**Param Reduction**		**GFLOP Reduction**		**mAP50 (%)**		**Speedup**
0% (Baseline)		0%		0%		76.1		1.00×
20%		18.7%		28.5%		75.9		1.12×
30%		27.5%		41.2%		75.8		1.17×
35%		36.3%		49.0%		75.6		1.20×
40%		42.8%		55.6%		74.8		1.24×
50%		53.1%		63.8%		73.2		1.29×

Note: The baseline (0% pruning) corresponds to the full unpruned YOLO-RMF model. All mAP values are averaged over 10 independent training runs with distinct random seeds, consistent with the statistical standard used in Table 1. Speedup is calculated relative to the baseline inference speed.

**Table 5:** Per-class detection performance on the NEU-DET dataset.

Defect Type	AP50 (%)	AP50-95 (%)	Recall	Precision	F1-Score	Instances
Crazing	56.4	30.9	0.601	0.587	0.593	60
Inclusion	84.1	44.0	0.897	0.873	0.884	60
Patches	93.5	49.5	0.997	0.972	0.984	60
Pitted Surface	81.7	43.8	0.871	0.851	0.860	60
Rolled-in Scale	55.0	30.4	0.586	0.570	0.577	60
Scratches	85.9	44.9	0.916	0.895	0.905	60
**mAP**	**76.1**	**40.6**	**0.812**	**0.791**	**0.801**	**360**

**Table 6:** Cross-dataset generalization performance.

Method	Train → Test	Source mAP50 on NEU-DET (%)	mAP50(%) on MVIT	mAP 50 Relative Drop
YOLOv5s	NEU → MVIT	71.5	42.5	40.5%
YOLOv8n	NEU → MVIT	73.1	41.7	42.9%
YOLOv8s	NEU → MVIT	74.5	42.8	42.5%
Defect-YOLO	NEU → MVIT	73.8	39.5	46.4%
**YOLO-RMF**	**NEU → MVIT**	**76.1**	**47.3**	**37.8%**

Note: The Relative Drop is calculated based on mAP50, defined as follows: $\mathrm{RelativeDrop}=\frac{{\mathrm{mAP}50}_{\mathrm{NEU}}-{\mathrm{mAP}50}_{\mathrm{MVIT}}}{{\mathrm{mAP}50}_{\mathrm{NEU}}}\times 100\%$ where mAP50_NEU_ is the model’s mAP50 on the NEU-DET test set (source domain, values consistent with Table 1), and mAP50_MVIT_ is the mAP50 on the MVIT test set (target domain) evaluated directly without fine-tuning.

**Table 7:** Robustness testing under perturbations.

Perturbation	Level	YOLOv8n mAP50	YOLO-RMF mAP50	Relative Gain
Gaussian Noise	σ = 0.05	62.3	65.8	+5.6%
Gaussian Noise	σ = 0.10	48.7	53.2	+9.2%
Salt-Pepper	5%	58.4	62.1	+6.3%
Brightness	−40%	55.2	59.7	+8.2%
Contrast	−30%	59.8	63.5	+6.2%
Gaussian Blur	σ = 2	61.5	65.2	+6.0%

**Table 8:** Deployment performance on Jetson AGX Orin.

Model	Precision	Inference Time (ms)	FPS	Memory Usage (MB)
YOLOv8n	FP32	7.2	139	485
YOLOv8n	FP16	4.8	208	342
YOLOv8n	INT8	4.6	217	228
YOLOv8s	FP32	10.5	95	865
YOLOv8s	FP16	7.8	128	592
YOLOv8s	INT8	5.6	178	385
YOLO-RMF	FP32	8.2	121	725
YOLO-RMF	FP16	6.8	147	512
YOLO-RMF	INT8	7.9	126	328
YOLO-RMF (Pruned)	FP16	5.9	169	345
YOLO-RMF (Pruned)	INT8	4.3	233	218

Note: FP32: 32-bit single-precision floating point; FP16: 16-bit half-precision floating point; INT8:8-bit integer quantization.

**Table 9:** End-to-end inspection system performance.

Component	Latency (ms)	Contribution
Image Capture	0.5	7.9%
Preprocessing	0.8	12.7%
Inference (INT8)	4.3	68.3%
Post-processing	0.7	11.1%
Total	6.3	100%

**Table 10:** Ablation module statistical significance.

Ablation Configuration	mAP50 Mean ± Std	mAP50-95 Mean ± Std	mAP50 95%CI
Baseline	73.13 ± 0.14	38.2 ± 0.08	[73.03, 73.23]
+RPRC2	74.38 ± 0.22 **	39.09 ± 0.15 **	[74.22, 74.54]
+CR2C_CR3C2	74.89 ± 0.36 **	39.30 ± 0.12 **	[74.63, 75.15]
+TDAA	75.45 ± 0.10 **	39.94 ± 0.11 **	[75.37, 75.52]
+MSF-FPN	75.78 ± 0.10 **	40.23 ± 0.12 **	[75.70, 75.85]
+FDetect Head (YOLO-RMF)	76.13 ± 0.16 **	40.60 ± 0.10 **	[76.01, 76.24]

Note: Results are reported as mean ± SD over 10 independent training runs. ** indicates a highly significant difference (Games-Howell adjusted *p* < 0.01) versus the preceding ablation configuration. Values in brackets are 95% confidence intervals.

**Table 11:** Algorithm statistical significance.

Comparison Method	mAP50 Mean ± Std	mAP50-95 Mean ± Std	95% CI
YOLO-RMF	76.13 ± 0.16	40.6 ± 0.10	[76.01, 76.24]
RetinaNet	70.01 ± 0.21 **	32.2 ± 0.13 **	[69.86, 70.16]
YOLOv5s	71.75 ± 0.27 **	36.7 ± 0.17 **	[71.56, 71.94]
YOLOv7-tiny	72.82 ± 0.49 **	37.5 ± 0.15 **	[72.47, 73.17]
YOLOv8n	72.82 ± 0.41 **	37.6 ± 0.18 **	[72.53, 73.11]
YOLOv8s	74.53 ± 0.20 **	39.4 ± 0.15 **	[74.38, 74.67]
Defect-YOLO	73.86 ± 0.20 **	38.7 ± 0.06 **	[73.72, 74.00]
Steel-DETR	75.36 ± 0.09 **	39.8 ± 0.10 **	[75.30, 75.42]
Faster R-CNN	72.40 ± 0.15 **	35.2 ± 0.10 **	[72.29, 72.51]

Note: Results are reported as mean ± SD over 10 independent training runs. ** indicates a highly significant difference (Dunn’s adjusted *p* < 0.01) versus YOLO-RMF. 95% confidence intervals for all detectors are provided in Table 11.

## Data Availability

Publicly available datasets were analyzed in this study. These data can be found here: http://faculty.neu.edu.cn/songkechen/zh_CN/zdylm/263270/list/index.htm (accessed on 14 January 2026) and https://github.com/clovermini/MVIT_metal_datasets (accessed on 16 May 2026).

## Abbreviations

The following abbreviations are used in this manuscript:

RPRC2	Reparameterized 2 × 2 convolution
TDAA	Two-dimensional axial attention
MSF-FPN	Multi-Scale Feature Fusion Pyramid Network
CSP	Cross Stage Partial
CR2C	CSP-RPR Bottleneck 2 Convolutions
CR3C	CSP-RPR-Bottleneck with 3 Convolutions
CR3C2	Dual CR3C
